# Portrait and factors associated with non-teaching school staff intention to deliver a comprehensive sexuality education program: an exploratory study

**DOI:** 10.3389/fpsyg.2026.1894282

**Published:** 2026-10-01

**Authors:** Julie Descheneaux, Joanne Otis

**Affiliations:** Department of Sexology, Université du Québec à Montréal, Montreal, QC, Canada

**Keywords:** comprehensive sex education, implementation, intention, psychosocial factors, school staff, sex education, sex educators

## Abstract

School team members such as psychoeducators and counselors can contribute to teach sexuality education curriculum to students, but little is known about their role. The purpose of this study was to develop a portrait of school team members who are solicited by teachers to teach sexuality education to their students, as well as their intention to do so and the factors associated with that intention. This descriptive exploratory study was conducted with 199 non-teaching school team members in two Quebec school service centers surveyed by online questionnaire between November 2018 and June 2019. Descriptive statistics and logistic regression analysis were used for data analysis. Overall, 32.7% of non-teaching staff were invited to provide sexuality education in teachers’ classrooms and nearly 65% of them intended to respond favorably to this invitation and teach sexuality education to students. There was a significant relationship between intention to teach sexuality education and age (OR = 0.883; *p* = 0.038), and social role (OR = 14.300; *p* = 0.032). The results of this study demonstrate that non-teaching staff can play a role in teaching sexuality education to students.

## 1 Introduction

Comprehensive school-based sexuality education (SE) programs are effective in reducing youth sexual health problems and improving their sexual well-being (Goldfarb and Lieberman, 2021; Loureiro et al., 2021; Yeung et al., 2017). These evidence-based programs begin at an early age and should be age- and culturally appropriate (UNESCO, 2015). Comprehensive SE addresses content in risk prevention and promotion of positive sexuality (e.g., prevention: sexual violence, HIV/AIDS, pregnancy, sex & gender stereotypes; promotion: sexual agency, consent, positive body image, romantic life). It addresses a multiplicity of themes that are complementary to biology, particularly socio-emotional learning, social norms or political/legal learning (e.g., the law on consent).

Several studies show that teachers lack the training to adequately deliver effective comprehensive SE (Cumper et al., 2024; Eisenberg et al., 2010; Goldman and Coleman, 2013). In this regard, many sensitive topics (e.g., abortion, LGBTQ+ inclusion) or emotional issues (e.g., managing emotions) tend to be sidelined by teachers (Oliveira et al., 2025). Community organizations, such as youth organizations, LGBTQ+ or sexual violence prevention organizations, as well as health professionals such as nurses or mental health professionals, are often called upon for their expertise in SE in schools (Moreira et al., 2023; Action Canada for Sexual Health and Rights, 2020; Arrington et al., 2018). Although their role in SE is less well known, non-teaching staff on the school team can also take on the role of sexuality educator to complement the teachers. Their collaboration is essential to achieving the mission of promoting sexual health in schools (Moreira et al., 2023). Psychoeducators, sexologists, special educators, psychologists, social workers, counselors, and remedial educators, for example, can teach a topic in which they specialize in order to implement complementary SE (Arrington et al., 2018; Loureiro et al., 2021). Their expertise allows them to take on the facilitation of SE activities on a few or many of the themes included in a comprehensive SE curriculum, depending on their original professional specialization. For example, psychoeducators may teach about romantic life and interpersonal conflict management. These health or educational professionals provide students with a range of services (clinical or educational) essential to their sexual health and well-being in addition to being recognized by students as a competent source of SE information (Akbari Kamrani and Yahya, 2016; Pavelová et al., 2021; Roberts et al., 2020). Their professional skills are useful for intervening in sensitive topics, such as sexual violence that may lead to classroom disclosures and legal obligations to report the situation to authorities (Eisenberg et al., 2013). In addition, they may have a positive attitude and high self-efficacy toward this task (Peskin et al., 2011).

While SE is delivered by teachers in a specific discipline in 24% of countries (e.g., health education in the United States) surveyed by UNESCO, 75% of countries integrate SE across the curriculum in other subjects (UNESCO, 2022). The province of Quebec in Canada has opted for a hybrid disciplinary and transdisciplinary mode (Descheneaux and Otis, 2023). Thus, the disciplines of ethics/ religious culture (or the new Culture and Citizenship in Quebec program starting in 2024) and science/technology must, respectively, teach SE contents (Pageau and Hirsch, 2025). Teaching of the comprehensive SE program is also a shared responsibility across disciplines since the reform of the Quebec school curriculum in 2000. All teachers are therefore expected to integrate SE into their teaching, even if none of them receive compulsory training in SE. To facilitate this teaching, mandatory sexuality education content was created in 2018. The content included eight themes that overlap from one year to another: Pregnancy & Birth; Sexual Growth & Body Image; Comprehensive View of Sexuality; Identity, Gender Stereotypes, and Social Norms; Sexual Assault & Violence; Emotional & Romantic Life; Sexual Behavior; Sexually Transmitted and Blood-borne Infections & Pregnancy (Ministère de l’Éducation et de l’Enseignement Supérieur, 2018). These themes can be taught in all school subjects, but each year, a minimum of 5 h of teaching at the elementary level and 10–15 h at the secondary level must be completed.

In the Quebec school model of shared responsibility, teachers can teach compulsory content on their own or call on collaborators to teach this content. These collaborators may come from outside the school (e.g., community based organizations), but they may also be school colleagues (e.g., non-teaching school staff). Each school has customized planning in which teachers, non-teaching school staff, and outside collaborators are engaged according to the availability of resources in the local context and their expertise (Otis et al., 2012). This planning is coordinated locally, but ultimately it is the teachers who invite non-teaching staff and community organizations into their classrooms. The curriculum is optionally delivered from preschool (4–5 years old) and then compulsorily in elementary school until Secondary V (6 to 17 years old).

Studies have shown that students tend not to prefer regular teachers to teach sexuality education content. For example, a meta-analysis reports that students prefer sex education specialists to teach this content, since teachers are less comfortable with it and teaching it jeopardizes confidentiality (Pound et al., 2016). Young et al. (2018) show that teaching this content by non-teachers (e.g., sexuality education specialists, school nurses, external agencies) has positive effects on sexual health indicators such as delayed sexual debut and condom use. A number of studies have shown that external school staff play a major role in the provision of services to cover sexuality education content from a comprehensive perspective, but few studies have focused on in-school collaborators (i.e., non-teaching staff). Health professionals, such as nurses, social workers and psychologists, are often mentioned as experts who can teach specialized content on sexual health, for example on puberty or HIV/AIDS prevention, even though their priority work in schools is generally to meet individual students (Bramhagen and Lundström, 2024; Pavelová et al., 2021; McCarty-Caplan and MacLaren, 2019). Some education professionals, particularly special educators, are mentioned as key players in teaching sexuality to students with special needs (e.g., students with disabilities) (Schaafsma et al., 2014), but their contribution to teaching the content of a formal sexuality education curriculum as part of classroom interventions remains little-known.

In short, it remains unclear whether teaching staff invite non-teaching staff to teach sexuality education content directly, or whether they only have a supporting role in creating an environment conducive to the teaching of sexuality education. In the Quebec model, as in many other curricula around the world, non-teaching staff are allies in delivering the SE program in order to achieve the health promotion mission (Action Canada for Sexual Health and Rights, 2020; Dickson et al., 2020). However, no studies to our knowledge have described non-teaching school staff who are invited to collaborate in delivering SE. This study attempts to develop a portrait of the non-teaching staff invited to deliver SE in schools and understand the factors associated with their intention to teach SE activities to students when solicited.

## 2 Materials and methods

### 2.1 Procedure and participants

This study is part of the project *Sexuality Education: A Portrait to Guide Action*, an intervention research project whose goal was to support the decision-making process of two school service centers whose mandate is to implement the Quebec SE curriculum. In this cross-sectional study, teachers, non-teaching staff and community partners were surveyed by questionnaires and interviews. For this study, only non-teaching school staff were surveyed via an online questionnaire.

Recruitment of participants was conducted in two phases, from November 2018 to January 2019 for the first center and from May to June 2019 for the second center. Participants received an email containing a link to the anonymous online survey on the Qualtrics platform, including the consent form. This email and the three reminders were sent by the SE school counselors using the official mailing lists provided by the school service centers. The participants were all employees of two French-language school service centers in suburban Montreal, Canada. It should be noted that nurses and doctors were not included in this study because they are not employees of school service centers. Of the 602 non-teaching school staff members targeted by the school service centers, 264 participated in the survey. Of these, 199 (75.4%) completed the questionnaire. The survey took approximately 52 min to complete. Four $100 gift certificates to local cultural centers were drawn for the participants of the overall study. The study was approved by the Université du Québec à Montréal (UQAM) student research ethics committee as well as by the health and social services center in the concerned region.

### 2.2 Theoretical model used in this study

The use of a theoretical model is recognized as a good practice to support program implementation (Bartholomew Eldredge et al., 2016). In the field of SE, theoretical models are mainly used to develop programs with students, but they are also useful for understanding the psychosocial factors associated with teaching SE. Using a theoretical model to understand the psychosocial factors influencing delivery of SE allows for a better understanding of who wants to engage in delivering comprehensive SE in the school environment. Ultimately, this will help guide the actions of managers to support the coordination and integration of SE services and ensure continuity in the teaching of sexuality education to guarantee a comprehensive approach to sexuality education, and, in this regard, ensure that socio-emotional content is taught on an equal footing with content related to prevention and health promotion.

For this study, we adopted Godin’s integrated theoretical framework, an adapted version of Ajzen’s (1991) Theory of planned behavior, in which expected behavior is primarily determined by the intention to perform that action. Intention is itself determined by a variety of psychosocial factors, most notably attitude, norm and perceived control. Attitude is the favorable or unfavorable perception of behavior adoption. Norm is the perception of whether or not others will adopt the behavior. Perception of control is the perception of being able to adopt the behavior or not. Past experience with the behavior is also an important variable in the model, but it is related more to the behavior itself than to intention. Similarly, socio-demographic variables, i.e., external variables, may mediate the link between intention and behavior, or influence intention indirectly, by modulating psychosocial factors. This integrative model has been used in several studies to explain, understand and predict the adoption of health-promoting behaviors, such as condom use or blood donation, and the adoption of health-promoting professional behaviors, such as those of nurses, dieticians and physicians. The psychosocial factors of Godin’s integrative theoretical model have also been shown to be effective in predicting the behaviors of healthcare professionals, particularly in contexts where the expected behavior is volitional (Godin et al., 2008). Its use has proved useful in our study, since non-teaching staff are voluntarily involved in teaching SE content within the Quebec model.

### 2.3 Measures

The questionnaire was developed following an 18-month participatory process informed by Ajzen’s recommendations for eliciting salient beliefs. The development process combined multiple qualitative data sources, including participant-observation meetings, unstructured interviews, an electronic questionnaire, and steering committee meetings. Findings from these activities were synthesized using Godin’s theoretical model to identify the salient beliefs underlying non-teaching school staff’s intention to deliver sexuality education and to operationalize the study constructs.

To establish content validity, the preliminary questionnaire underwent several rounds of expert and stakeholder review. First, a panel of six researchers with expertise in sexuality education and implementation independently reviewed the instrument and provided written recommendations regarding item relevance, clarity, and comprehensiveness. Second, the revised version was reviewed by five school professionals (three health professionals and two educational allied professionals) from School Service Centers not involved in the study to assess clarity, comprehensibility, and practical relevance. Finally, additional refinements were made following discussions with the research steering committee and representatives from professional unions. These iterative revisions ensured that the questionnaire adequately reflected both the theoretical constructs and the realities of school-based SE practice.

The survey measured respondents’ invitation in teacher’s classroom to teach SE, intention to deliver SE when they are invited (dependent variable) as well as psychosocial factors (predictors) and sociodemographic variables. Table 1 provides a complete overview of variables in the survey including a description of the main question, number of scale items, examples of items, internal consistency, original and modified measures.

**Table 1:** Survey measures.

Variable	Description or main question	Scale			Original measure [score range]	Modified measure
		No of items	Example of items	Internal consistancy		
Filter variable						
Invitation in a teacher’s classroom to teach SE	During the next year, do you think you could be invited to a teacher’s classroom to teach SE?	—	—	—	Yes; maybe; no	Dichotomized Yes and maybe = 1 No = 0
Dependent variable						
Intention to deliver SE	How much do you agree with the following statements? During the next year, I intend to teach SE to students if I am invited by teachers in their classroom	—		—	1 (strongly disagree)–7 (strongly agree)	Dichotomized 1 to 4 = no 5 to 7 = yes
Independent variable						
Past experience	Last year, did you teach SE activities?	—	—	—	Yes; no	NA
Cognitive dimension of attitude	Regarding the SE concepts to be taught next year, how much do you agree with the following potential effects on students?	10	Students’ sexual health will be improved if I teach them SE	*α* = 0.86	1 (strongly disagree)–7 (strongly agree)	Dichotomized 1 to 4 = no 5 to 7 = yes
Affective dimension of attitude	Regarding the SE concepts to be taught next year, how much do you agree with the following potential effects on yourself?	12	I will enjoy doing my job if I teach SE	*α* = 0.77	1 (strongly disagree)–7 (strongly agree)	Dichotomized 1 to 4 = no 5 to 7 = yes
Anticipated regret	How much do you agree with the following statements about sexuality education to be taught to students next year?	2	If I do not teach SE to students, I will feel regret	0.84	1 (strongly disagree)–7 (strongly agree)	Dichotomized 1 to 4 = no 5 to 7 = yes
Subjective norm	[Normative beliefs] How much do you agree with the following statements? Regarding SE to be taught to students next year…[multiplied by motivation to conform] To what extent do you feel influenced by these people or groups of people regarding your decision making about teaching SE concepts?	9	“Students will be satisfied that sexuality education is taught to them”; “My students’ parents will endorse my initiative”; “My manager will support my initiative” “Students”;” Students’ parents”; “My manager”	0.92	1 (strongly disagree)–7 (strongly agree) [1–49]	NA
Descriptive norm	In your opinion, of all the allied professionals you know in your school district, how many of them (%) teach SE	—	—	—	0–100	Dichotomized 0 to 50 = no 51 to 100 = yes
Moral norm	How much do you agree with the following statements about SE to be taught to students next year?	3	Teaching SE to students is a matter of duty that I find self-fulfilling	0.92	1 (strongly disagree)–7 (strongly agree)	Dichotomized 1 to 4 = no 5 to 7 = yes
Social role	How much do you agree with the following statements about SE to be taught to students next year?	4	Teaching students about SE is part of an allied professional’s job	0.88	1 (strongly disagree)–7 (strongly agree)	Dichotomized 1 to 4 = no 5 to 7 = yes
Feeling comfortable with teaching the curriculum	How easy will it be for you to teach the following topics to students next year?	8	“Pregnancy & Birth,” “Sexual Assault & Violence,” “Sexual Behavior” [themes include the 8 themes of the Quebec curriculum]	0.95	1 (strongly disagree)–7 (strongly agree)	NA
Self-efficacy in sexual health education	Derived from Self-efficacy in Health Education Role scale (Plouffe et al., 2011)	9	I would be able to adapt the Minister of Education’s SE topics to students’ needs”	0.94	1 (strongly disagree)–7 (strongly agree)	Dichotomized 1 to 4 = no 5 to 7 = yes
Self-efficacy with working in partnership	Derived from Self-efficacy with working in partnership for Health Education scale (Plouffe et al., 2011)	10	I feel that I would be able to participate with other stakeholders in setting common goals	0.94	1 (strongly disagree)–7 (strongly agree)	Dichotomized 1 to 4 = no 5 to 7 = yes
Facilitating factors	How much do you agree with each of the following statements? During the next year, it will be easier for me.	17	“If my school staff is mobilized,” “If a resource person supports me” [all facilitators were about support in the school environment]	0.94	1 (strongly disagree)–7 (strongly agree)	Dichotomized 1 to 4 = no 5 to 7 = yes

As recommended by Eisenga et al. (2013), we calculated Cronbach’s alpha for multiple-items scales and Spearman-Brown Coefficient for two-items scales.

### 2.4 Statistical analysis

Univariate and bivariate analyses were performed on the factors associated with intention (predictors) and on intention to deliver SE (dependent variable). Logistic regression was then performed on significant variables to identify factors associated with intention of delivering SE. Variable selection for the multivariable logistic regression was guided by Godin’s integrated model of health behavior. All theoretical constructs specified by the model were considered for inclusion. When multiple variables operationalized the same construct, bivariate analyses were used to identify the indicator most strongly associated with the outcome. This strategy was adopted to avoid redundancy among predictors and to minimize potential multicollinearity while maintaining representation of each theoretical construct in the final model. Significant variables at *p* < 0.05 in the bivariate analysis were included in the logistic regression and variables were significant at the same level in the logistic regression. Analyses were conducted using the statistical software SPSS, version 27. The basic assumptions of no multicollinearity and independence of residuals were respected. The tolerance indicators were greater than 0.10 and the VIF values were lower than 10. Examination of the residuals showed that they were normally distributed (Pallant, 2013). Model fit was evaluated using the Nagelkerke pseudo-*R*^2^.

## 3 Results

As shown in Table 2, a total of 199 non-teaching staff members were included in the study of which one hundred and seventy-five (87.9%) were female and ninety-eight (44.2%) were university graduates. The mean age of the participants was 41.3 years (SD 8.9). Ninety-five people (47.7%) have a full-time job while the rest work part-time. More than 47% (47.2%) are employed at the primary level, 39.7% at the secondary level while 13.1% work in both cycles of study. Of the participants, 42.2% are health care professionals, 36.7% are educational allies and 21.1% are administrative staff. Eighty people (40.2%) received one hour or more of training in SE. Ninety-five people (47.7%) were at least a bit familiar with the new curriculum.

**Table 2:** Sample description (*n* = 199).

Variables	Participants	
	*n*	% or SD
Gender		
Men	24	12.1
Women	175	87.9
Education		
University (under-graduated and graduated degree)	88	44.2
Other (secondary, college diploma)	111	55.8
Mean age (in years)	41.3	8.9
Work status		
Full-time (100%)	95	47.7
Part-time (0–99%)	104	52.3
Working level		
Primary school	94	47.2
Secondary school	79	39.7
Both primary and secondary school	26	13.1
Profession		
Health professionals (special need educators, psychoeducator, psychologist, etc.)	84	42.2
Educational allied (counselor, remedial teacher, student supervisor, daycare service, practical work and laboratory, etc.)	73	36.7
Administration staff	42	21.1
Received training in SE (at school or at work)		
At least 1 h	80	40.2
None	119	59.8
Being familiar with the new SE curriculum		
At least a little bit	95	47.7
No	104	52.3

### 3.1 Descriptive statistics of school staff invitation in teachers’ classroom and intention to deliver comprehensive sex education (when invited)

The invitation of non-teaching school staff to provide comprehensive SE by their type of profession is described in Table 3. Overall, 32.7% of non-teaching staff are invited to provide SE in teachers’ classrooms; 50% of health professionals are invited compared to 31.5% for educational allies while no administrative staff are invited to provide SE in classrooms (*p* < 0.001). Table 3 also describes the intention of non-teaching staff when they are invited into classrooms. Nearly 65% (64.6%) of invited staff intend to respond favorably to this invitation and teach SE to students, regardless of profession. Results show that specialized educators are the most likely non-teaching school staff to be called upon to teach SE. Also, unexpected professions among educational allies, such as laboratory technicians, can contribute to the delivery of SE.

**Table 3:** School staff invitation in teachers’ classroom and intention to deliver sex education (when being invited in teacher’s classroom), by profession (*n* = 199).

Profession	Invitation in teachers’ classroom			Intention to deliver sex education (when being invited in teacher’s classroom)		
	Yes or may be *n* (%)	No *n* (%)	*p*-value	Yes *n* (%)	No *n* (%)	*p*-value
Health professionals	42 (50.0)	42 (50.0)	<0.001	27 (64.3)	15 (35.7)	0.940
Special need educator	34	20		20	14	
Psychoeducator	6	4		5	1	
Psychologist	2	8		2	0	
Speech language pathologist	0	6		—	—	
Others	0	4		—	—	
Educational allied	23 (31.5)	50 (68.5)		15 (65.2)	8 (34.8)	
Counselor (for students or organizational)	9	14		7	2	
Remedial teacher	3	14		1	2	
Student supervisor	3	7		3	0	
Daycare service	0	9		—	—	
Practical work and laboratory	5	3		2	3	
Others	3	3		2	1	
Administration staff	0 (0)	42 (100.0)		—	—	
Secretary	0	36		—	—	
Others	0	6		—	—	
Total	65 (32.7)	134 (67.3)		42 (64.6)	23 (35.4)	

### 3.2 Bivariate analysis and regression model examining factors associated with intention to teach sex education, by health professionals and educational allies

Associations between psychosocial variables, including sociodemographic variables, and intention are presented in Table 4. In terms of socio-demographics, a higher proportion of those with previous SE training and knowledge of the new SE curriculum intend to teach SE. Similarly, a higher proportion of those who intend to teach SE have high cognitive attitudes, high affective attitudes, and high anticipated regret if they do not teach SE when invited to do so. Those who intend to teach SE also have a higher subjective norm, and a higher proportion have a high moral norm and a high social role. Finally, they also have greater comfort in teaching curriculum topics and are more likely to have a high sense of self-efficacy in teaching sexual health and to perceive facilitators positively.

**Table 4:** Bivariate analysis of factors associate with intention to teach sex education when being invited in teacher’s classroom (*n* = 65).

Variables	Intention to teach sex education when being invited in teacher’s classroom			
	Total *n* = 65 (%, SD)	No *n* = 23 (%, SD)	Yes *n* = 42 (%, SD)	*p*-value
Sociodemographic				
Gender				
Men	6 (9.2)	2 (8.7)	4 (9.5)	0.643
Women	59 (90.8)	21 (91.3)	38 (90.5)	
Mean age	41.3 (7.8)	42.5 (6.2)	40.7 (8.6)	0.358
Education				
University	31 (47.7)	8 (34.8)	23 (54.8)	0.123
Other (college diploma, secondary)	34 (52.3)	15 (65.2)	19 (45.2)	
Working				
At primary or secondary school	56 (86.2)	22 (95.7)	34 (81.0)	0.148
At both (primary and secondary schools)	9 (13.8)	1 (4.3)	8 (19.0)	
Profession				
Health professionals	42 (64.6)	15 (65.2)	27 (64.3)	0.940
Educational allied	23 (35.4)	8 (34.8)	15 (35.7)	
Received training in SE (at school or at work)				
No	**28 (43.1)**	**14 (60.9)**	**14 (33.3)**	**0.032**
Yes	**37 (56.9)**	**9 (39.1)**	**28 (66.7)**	
Being familiar with the new SE curriculum				
No	**23 (35.4)**	**12 (52.2)**	**11 (26.2)**	**0.036**
Yes	**42 (64.6)**	**11 (47.8)**	**31 (73.8)**	
Past experience				
No	47 (72.3)	19 (82.6)	28 (66.7)	0.170
Yes	18 (27.7)	4 (17.4)	14 (33.3)	
Attitude				
Cognitive dimension of attitude				
No	**6 (9.2)**	**5 (21.7)**	**1 (2.4)**	**0.018**
Yes	**59 (90.8)**	**18 (78.3)**	**41 (97.6)**	
Affective dimension of attitude				
No	**12 (18.5)**	**8 (34.8)**	**4 (9.5)**	**0.016**
Yes	**53 (81.5)**	**15 (62.2)**	**38 (90.5)**	
Anticipated regret				
No	**38 (58.5)**	**19 (82.6)**	**15 (39.5)**	**0.003**
Yes	**27 (41.5)**	**4 (17.4)**	**23 (60.5)**	
Norms				
Subjective norm	**27.9 (1.1)**	**22.7 (1.9)**	**30.7 (1.1)**	**<0.001**
Descriptive norm				
No	57 (87.7)	22 (95.7)	35 (83.3)	0.146
Yes	8 (12.3)	1 (4.3)	7 (16.7)	
Moral norm				
No	**22 (33.8)**	**15 (65.2)**	**7 (16.7)**	**<0.001**
Yes	**43 (66.2)**	**8 (34.8)**	**35 (83.3)**	
Social role				
No	**15 (23.1)**	**12 (52.2)**	**3 (7.1)**	**<0.001**
Yes	**50 (76.9)**	**11 (47.8)**	**39 (92.9)**	
Perception of control				
Feeling comfortable teaching the curriculum	**4.8 (0.2)**	**4.0 (0.4)**	**5.3 (0.2)**	**0.002**
Self-efficacy in sexual health education				
No	**25 (38.5)**	**13 (56.3)**	**12 (28.6)**	**0.027**
Yes	**40 (61.5)**	**10 (43.5)**	**30 (71.4)**	
Self-efficacy with working in partnership				
No	24 (36.9)	11 (47.8)	13 (31.0)	0.178
Yes	41 (63.1)	12 (52.2)	29 (69.0)	
Facilitators				
No	**7 (10.8)**	**5 (21.7)**	**2 (4.8)**	**0.048**
Yes	**58 (89.2)**	**18 (78.3)**	**40 (95.2)**	

Bold text indicates that the analyses are statistically significant at < 0.05 level.

The final logistic regression model examining non-teaching school staff’s intention to deliver SE yielded a Nagelkerke pseudo-*R*^2^ of 0.656. In this model, significant factors associated with intention was age (OR = 0.883; *p* = 0.038) and social role (OR = 14.300; *p* = 0.032). Table 5 details the results.

**Table 5:** Logistic regression model examining factors of the intention to deliver comprehensive sex education, by health professional and educational allied being invited in teacher’s classroom (*n* = 65).

Variables indépendantes	Model 1			Model 2		
	OR	OR 95% CI	*p*-value	OR	OR 95% CI	*p*-value
Sociodemographic						
Gender						
Men	Ref.			Ref.		
Women	0.642	0.086–4.820	0.657	0.728	0.033–16.092	0.840
Age	0.947	0.870–1.031	0.210	**0.883**	**0.785–0.093**	**0.038**
Education						
Other (college diploma, secondary)	Ref.			Ref.		
University	2.259	0.585–8.732	0.237	1.721	0.291–15.844	0.605
Working						
At primary or secondary school	Ref.			Ref.		
At primary and secondary schools	5.905	0.527–66.150	0.150	0.626	0.030–13.234	0.764
Profession						
Health professionals	Ref.			Ref.		
Educational allied	1.197	0.322–4.448	0.788	0.105	0.007–1.528	0.099
Received training in SE (at school or at work)						
No	Ref.			Ref.		
Yes	2.844	0.843–9.591	0.092	3.128	0.479–20.417	0.233
Being familiar with the new SE curriculum						
No	Ref.			Ref.		
Yes	3.023	0.903–10.116	0.073	2.963	0.419–20.971	0.277
Attitude, norms, perception of control						
Cognitive dimension of attitude				7.456	0.295–188.289	0.223
Affective dimension of attitude				0.588	0.028–12.442	0.733
Anticipated regret				2.658	0.382–18.509	0.324
Subjective norm				1.082	0.953–1.227	0.223
Moral norm				1.895	0.179–20.056	0.595
Social role				**14.300**	**1.252–163.360**	**0.032**
Feeling comfortable teaching the curriculum				1.656	0.808–3.397	0.169
Self-efficacy in sexual health education				1.441	0.134–15.479	0.763
Facilitators				1.790	0.043–74.914	0.760
*R*^2^ de Nagelkerke	0.270			0.656		

OR, odds ratio; 95% CI, 95% confidence interval; Ref: reference group. *p < 0.05; **p < 0.01; ***p < 0.001. Bold text indicates that the analyses are statistically significant at < 0.05 level.

## 4 Discussion

This study attempts to document the profile of non-teaching staff invited by teachers to teach SE in their classrooms, to better understand their intention to teach SE when invited to do so and to identify the factors associated with their intention. This exploratory study is the first to our knowledge to investigate this area of research.

This study shows that non-teaching staff have an important role to play in SE, but that their involvement varies greatly from one professional body to another. Health professionals (e.g., psychoeducator, psychologist; see Table 3) are the most frequently asked to provide SE in the classroom, with 50.0% of them reporting that they could be invited into teachers’ classrooms to teach activities, compared to only 31.5% of educational allies (e.g., counselors, remedial teachers; see Table 3) and, not surprisingly, none of the administrative staff. It is clear that the expertise of health professionals is an important anchor of the comprehensive SE program for students in the school environment as suggested by the Canadian Guidelines for Sexual Health Education (SIECCAN, 2019), but it is not limited to nurses. Educational allies like counselors, remedial teachers, students supervisors and practical work and laboratory technicians are also actors called upon to provide SE instruction, but they could be further engaged. The study shows that they may be just as likely as health professionals to respond positively when called upon to provide SE, since their level of intention to teach SE is similar. However, their contribution to SE seems to be largely unknown in the scientific literature and deserves to be investigated.

This study shows that there are a variety of professions involved in SE. Concerning the solicitation of specialized educators, this is a positive sign regarding the implementation of the program. Special educators in Quebec are on the front line of support for students with disabilities, such as physical or neurodevelopmental impairments (e.g., autism spectrum disorder, intellectual disability). Several studies show the extent of the sex-educational needs of students with disabilities, particularly because they are more at risk of experiencing and perpetuating sexual violence, but also because their social isolation and the stigmatization of their sexuality often lead them to lack basic information about romantic life, sexual conduct or sexual health for example (Borawska Charko et al., 2017; Grove et al., 2018; Michielsen and Brockschmidt, 2021). These specialized educators have an important role to play in informing students with disabilities, especially in the context where, as our results point out, remedial educators who intervene as their main teacher in the province of Quebec, feel less involved in SE. Unexpected professions can also contribute to the delivery of SE in a model where sexuality education content is taught by various sex educators to ensure a comprehensive approach. These findings lead us to recommend specialized training for special educators and educational allies in order to engage them more fully as sexuality educators. They should be exposed during their initial training to SE as other studies have suggested (Jiménez-Ríos et al., 2023). As specialists in human sexuality, sexologists could also have a key role in supporting their professional development since they are trained in these issues.

Overall, this study shows that there is a wide variety of professions involved in SE at the local level as other studies have shown (Barriuso-Ortega et al., 2024). While the contribution of school health professionals in the Quebec context seems to be concentrated in special educators, psychoeducators and, to a lesser extent, psychologists, we have identified seven different professions that are involved among educational allies. These results are surprising, given that these professions are not associated with sexuality education in popular belief. Nor are they trained to master the content to be taught in sexuality education. Nevertheless, their involvement in sexuality education could testify, as other studies suggest, that the educational attitude (e.g., open-minded attitude, youth-centered approach) is sometimes just as, if not more, important than mastery of content at a theoretical level (Kenneally, 2026). It should also be pointed out that Quebec’s sexuality education program encompasses specialized content (e.g., sexual health; prevention of love and sexual violence), but also generic content (e.g., globality of human sexuality; love life) that could be more accessible to this variety of professions. In this sense, several content related to the socio-emotional domain might be easier to teach. That said, it remains difficult to fully understand the roles played by this variety of profession in the teaching of sexuality education under a shared responsibility model. This multiplicity could also be a reflection of a lack of resources at the local level to teach the program or, on the contrary, a sign that schools are very committed and a mobilization is taking place among all the staff in the school in line with health promotion approaches such as the Whole School, Whole Community and Whole Child model (Lewallen et al., 2015). This highlights the need to allocate resources to adequately coordinate service provision to deliver comprehensive SE (Keogh et al., 2018; Ollis and Harrison, 2016; Otis et al., 2012), but also to form multidisciplinary teams in schools to support the implementation of sexuality education, as suggested by the systematic literature review by Barriuso-Ortega et al. (2024).

In addition, we find that further research is needed to better understand the factors that influence this differential solicitation within the same job category, i.e., among health professionals and, especially, among educational allies. Beyond professional affiliation, personal (e.g., motivation to teach SE), organizational (e.g., lack of staff) or territorial (e.g., socioeconomic deprivation index of the neighborhood) factors could explain this difference in demand.

Second, approximately 65% of health professionals and educational allies intend to respond favorably to teachers’ invitations to provide SE in the inviting teachers’ classrooms. This similar finding for both professional groups is surprising given that the literature on non-teacher sexuality educators focuses primarily on the input of health professionals (Psarouli et al., 2026; Barriuso-Ortega et al., 2024; Pavelová et al., 2021; Borawski et al., 2015). This further supports the idea that the role of educational allies in SE should be emphasized, and requires a better understanding to do so.

The results further suggest that variables related to the attitude, norm, and perceived control construct are associated with non-teachers’ intention to teach SE, but not past experience. A lack of sensitivity of the past experience measure in the study could explain this result although it also confirms Godin’s model that past experience influences behavior, but not directly the intention to perform the behavior (Godin and Vézina-Im, 2012). The results further demonstrate that social role (identification with others who have certain characteristics - here rooted in the professional role at work) is a key determinant associated with the intention to teach SE among non-teaching school staff. Indeed, the belief that one’s profession has a role to play in SE is crucial in forming a favorable intention to teach SE among the non-teaching staff in this study. Valuing the contribution of multiple professions in SE is consistent with the multidimensional perspective of comprehensive SE and health promotion (Unis et al., 2021), but may be counterintuitive in the Quebec context of professionalization of sexology when many may tend to believe that only sexologists have the skills to intervene in the field of sexuality. Studies should attempt to better understand the professional contributions in SE and the issues of interprofessional collaboration.

Other attitude, norm, and perceived control variables are associated with non-teaching staff’s intention to teach SE. The cognitive (advantages and disadvantages of engaging in the behavior for students, i.e., beliefs and knowledge of the effects of teaching SE) and affective (advantages and disadvantages of engaging in the behavior for oneself, i.e., feelings and reactions elicited) dimensions of attitude as well as anticipated regret about not engaging in the behavior are three attitudinal variables associated with intention to teach SE. The study shows that a high proportion of non-teaching staff have a favorable attitude toward SE, particularly in the cognitive and affective dimensions, confirming the high social acceptability of teaching SE in schools in Canada (Wood et al., 2021). To our knowledge, this study is the first to demonstrate an association between anticipated regret and intention to teach SE among non-teaching school staff. Subjective norm (perceived sense of approval or disapproval from those around me when I perform the behavior) and moral norm (belief that the behavior is consistent with my sense of social responsibility) are, in addition to social role, associated in this study with intention to teach SE. Given that teaching SE is a politically sensitive behavior, the perceived pressure of the social environment, including its internalized form, appears to be factors that influence intention. Finally, perceived control demonstrates, similar to other studies among teachers, that comfort with teaching SE curriculum topics and perceived facilitators to perform the behavior are associated with intention to teach SE (Cohen et al., 2012; Mkumbo, 2012). The results further demonstrate that self-efficacy to teach sexual health, a health education scale adapted for this study (see Plouffe et al., 2011), is associated with intention to teach SE. This implies that beyond being knowledgeable about sensitive topics, non-teaching school staff should integrate the SE cross-curricular program into their daily tasks (e.g., adapting the curriculum objectives to their students).

### 4.1 Limitations

This study has several limitations and should be considered exploratory. First, the cross-sectional design precludes establishing temporal or causal relationships among the variables examined. Although the observed associations are consistent with Godin’s integrated model of health behavior, they should not be interpreted as demonstrating causal relationships between psychosocial factors and non-teaching school staff’s intention to deliver SE. Longitudinal studies are needed to examine the temporal relationships proposed by the theoretical model and to determine whether these factors predict subsequent participation in SE activities.

Second, the study relied on self-reported behavioral intention rather than actual delivery of SE. Although behavioral intention is considered the most proximal determinant of behavior within Godin’s integrated model, intention does not necessarily translate into action. In school settings, organizational constraints, competing professional responsibilities, limited opportunities to deliver SE, or lack of administrative support may prevent school staff from acting on their intentions. Future research should therefore examine whether behavioral intention predicts actual participation in SE and identify contextual factors that facilitate or hinder the translation of intention into behavior.

Third, the sample was relatively small and composed of volunteers recruited from two French-language School Service Centers in suburban Montreal, making the study susceptible to self-selection bias and limiting the transferability of the findings. The specific organizational structure of Quebec’s education system and the roles assigned to non-teaching school staff may differ from those in other educational jurisdictions. Consequently, caution should be exercised when transferring these findings to other educational contexts. The survey’s length may also have contributed to participant attrition. Comparisons between participants who completed the survey and those who withdrew indicated that only moral norm differed significantly between the two groups, with none of the participants who withdrew reporting high moral norm scores.

Fourth, although the questionnaire was developed through a rigorous theory-informed participatory process and demonstrated satisfactory internal consistency, additional psychometric evaluation is required. Future studies should further examine its construct validity, temporal stability, and performance in larger and more diverse samples.

Finally, the relatively small sample size increased the risk of model overfitting given the number of predictors included in the logistic regression analysis. Replication using larger and more diverse samples is therefore needed to confirm the present findings. In addition, nurses were not included because they are not employed by Quebec School Service Centers. Consequently, the findings should not be generalized to all professional groups involved in school-based SE.

Despite these limitations, this exploratory study provides valuable insights into the implementation of school-based SE by identifying factors associated with non-teaching school staff’s intention to deliver SE. The findings also contribute to a better understanding of the organizational and psychosocial conditions that may support the implementation of comprehensive SE in school settings.

## 5 Conclusion

The study demonstrates the importance of valuing the diversity of professional bodies in SE and developing interprofessional skills in order to maintain a comprehensive SE program in schools. Non-teaching school staff can play a key role in SE, both in supporting the provision of clinical services regarding sexual health needs of students at school (e.g., counseling, referral to sexually transmitted and blood-borne infections screening or abortion resources), but also in teaching the large range of topics covered in the comprehensive SE curriculum. In this regard, it seems that the school’s mission of prevention and promotion of sexual health and well-being is becoming more and more meaningful to those involved in education. In order to value this professional diversity, it is important to highlight the role of non-teaching school staff in SE and the sense of belonging to their profession.

## Data Availability

The raw data supporting the conclusions of this article will be made available by the authors, without undue reservation.
